# Outcomes of Gastrointestinal Bleeding During the COVID-19 Pandemic

**DOI:** 10.1016/j.gastha.2022.02.006

**Published:** 2022-02-11

**Authors:** R.W. Rehana, H. Fahad, O. Sadiq, J. Schairer

**Affiliations:** 1 Department of Internal Medicine, Henry Ford Macomb Hospital, Clinton Township, Michigan; 2 Department of Gastroenterology, Henry Ford Hospital, Detroit, Michigan

The novel circumstances during the pandemic have led to many challenges for clinicians and patients, which includes cancellation of procedures, policy regulations, and the reluctance of patients to seek medical attention potentially leading to adverse outcomes.^1^^,^^2^ Although outpatient appointments and elective procedures are less likely to affect short-term patient outcomes, the delay in seeking care for medical emergencies can be devastating. Gastrointestinal bleeding (GIB) is a commonly encountered emergency. Its severity varies from mild to severe and can be life-threatening.^3^ Patients with GIB require early resuscitation and possible intervention.^4^ The aim of this study was to investigate differences in the outcomes of hospitalized patients who encountered GIB during the initial peak of Coronavirus 2019 (COVID-19) pandemic in Michigan, USA, during the month of March 2020, compared to the year prior.

In this retrospective cohort study, hospitalized patients who were found to have GIB, at our institution during the first 2 weeks of March 2019 and March 2020, were identified using ICD-10 codes for GIB (K92.1 melena, K92.0 hematemesis, K92.2 gastrointestinal bleed, K25.9 gastric ulcer without bleed, K57.21 diverticular bleed, K62.5 hematochezia, K31.811 angiodysplasia, I85.01 esophageal variceal bleed, K29.61 other gastritis with bleeding, K29.81 duodenitis with bleeding). Patients older than 18 years who were noted to have GIB during hospitalization were included. Patients who were discharged from the emergency department or had a prior history of GIB without evidence of current bleeding were also excluded. Study time was decided based on Michigan’s initial COVID-19 surge and correlated with the mandate for state-wide restrictions. Institutional electronic medical records were retrospectively reviewed to collect data. The diagnosis of GIB was identified based on the clinical evidence of gross hemorrhage such as hematemesis, coffee-ground emesis, hematochezia, and/ormelena and/or supported by an assessment of nadir hemoglobin (Hb) during index hospitalization compared to baseline Hb and/or confirmed by diagnostic imaging such as computed tomography (CT), CT angiography, capsule endoscopy, tagged red blood cell scan, esophagogastroduodenoscopy (EGD), or colonoscopy. Variables of interest included age, gender, ethnicity, cirrhosis, peptic ulcer disease, prior GIB, coagulopathy, anticoagulation, date of COVID-19 swab, time to COVID-19 results, time to intervention, type of intervention (EGD, colonoscopy, therapeutic angiography with embolization), hemodynamic instability (tachycardia, hypotension, orthostasis, dizziness, light-headedness, syncope, dyspnea), admission Hb, nadir Hb during hospitalization, and mortality. Analysis was conducted with the Welch T-test and Pearson's chi-square test utilizing R, version 4.1.0.

A total of 308 patients were identified on the initial search. Of these, 89 patients met the inclusion criteria in 2019 and 76 patients met the inclusion criteria in 2020. Of the 2020 cohort, 88.2% tested positive for COVID-19. When compared with 2019, patients during COVID-19 pandemic in 2020 were significantly older (years) (62.1 vs 68.3, *P* < .01), required longer hospital length of stay (days) (11.3 vs 20.9, *P* < .01; Table 1), had higher rate of mortality (12.5% vs 36%, *P* < .01), were less likely to have a history of cirrhosis (25.8% vs 6.6%, *P* < .01), presented with a higher Hb (mL/dL) (9.9 vs 11.9, *P* < .001), and were less likely to undergo EGD (58.4% vs 13.2%, *P* < .001) or colonoscopy (21.6% vs 4%, *P* < .01). In 2019, 7 patients had an active source of bleeding identified on CT angiography and underwent interventional radiology (IR)–guided therapy (6 patients had coil embolization, 1 had transjugular intrahepatic portosystemic shunt placement) compared with a single patient who underwent IR-guided embolization in 2020. There was no significant difference between baseline Hb and nadir Hb (*P* > .05). The most common causes of primary death in 2019 were decompensated liver failure (27.3%), followed by septic shock (18%) and cardiogenic shock (18%), compared to 2020 deaths, which were COVID-19 respiratory failure (67.9%), hemorrhagic shock (11.1%), and cardiogenic shock (7%; Table 2).

**Table 1:** Demographic and Laboratory Parameters

Demographic and laboratory parameters	Year 2019	Year 2020
Male (%)	51.68	57.89
Age (y ± SD)	62.12 (±15.45)	68.25 (±11.66)
Admission hemoglobin (mL/dL ± SD)	9.87 (±2.52)	11.85 (±2.77)
Length of stay (d ± SD)	11 (±21.79)	20.17 (±18.93)
Lowest hemoglobin reached (mL/dL ± SD)	8.00 (±2.14)	8.18 (±2.44)
Baseline hemoglobin (mL/dL ± SD)	11.22 (±2.46)	11.92 (±2.18)
Time to COVID-19 result (d ± SD)	n/a	1.1 (±0.38)

**Table 2:** Primary Cause of Death

Primary cause of death	Year 2019	Year 2020
Cardiac arrest	1 (9.1%)	2 (7.4%)
Hemorrhagic shock	1 (9.1%)	3 (11.1%)
Intracranial hemorrhage	0	1 (3.7%)
Renal failure	0	1 (3.7%)
COVID-19 respiratory failure	0	19 (70.4%)
Cardiogenic shock	2 (18.2%)	0
Decompensated liver failure	3 (27.3%)	0
Septic shock	2 (18.2%)	0
Multiorgan failure	1 (9.1%)	0
Shock liver	1 (9.1%)	0
Undifferentiated shock	0	1 (3.7%)
Total deaths	11	27

Our study highlights the fact that most patients who presented for GIB during the 2020 study period also tested positive for COVID-19. Additionally, despite presenting with a higher Hb on admission, patients during the COVID-19 pandemic had a higher rate of mortality, with the most common cause of death being respiratory failure from COVID-19. Our results also suggested that patients with GIB were less likely to undergo endoscopic evaluation during the pandemic; however, this was not associated with increased mortality secondary to hemorrhage. Given the retrospective nature of our study, it is difficult to say whether decisions regarding endoscopic evaluation were influenced by patient-related factors, such as hematologic derangements, COVID-19–related coagulopathy, and/or cardiopulmonary instability, making endoscopy high risk. Moreover, institution- and personnel-related factors, such as strict COVID-19 isolation precautions aimed at reducing aerosolizing procedures, duration of screening COVID-19 swab results, or clinical assessment, suggested that GIB was not the primary cause of patient demise.

While endoscopic evaluation is the gold standard for diagnosis and treatment of GIB,^5^ factors such as COVID-19 coagulopathy and cardiopulmonary compromise can limit our ability to perform a safe procedure and necessitate other treatment modalities.^6^ Based on our findings, we propose a multidisciplinary approach while making critical decisions about treatment of GIB during the COVID-19 pandemic.^7^ Discussion should involve the intensive care unit team, IR services, as well as surgical services.
